# Bayesian estimation of the time-varying reproduction number for pulmonary tuberculosis in Iran: A registry-based study from 2018 to 2022 using new smear-positive cases

**DOI:** 10.1016/j.idm.2024.05.003

**Published:** 2024-05-10

**Authors:** Maryam Rastegar, Eisa Nazar, Mahshid Nasehi, Saeed Sharafi, Vahid Fakoor, Mohammad Taghi Shakeri

**Affiliations:** a Department of Biostatistics, School of Health, Mashhad University of Medical Sciences, Mashhad, Iran; b Orthopedic Research Center, Mazandaran University of Medical Sciences, Sari, Iran; c Centre for Communicable Diseases Control, Ministry of Health and Medical Education, Tehran, Iran; d Department of Statistics, Faculty of Mathematical Sciences, Ferdowsi University of Mashhad, Mashhad, Iran; e Social Determinants of Health Research Center, Mashhad University of Medical Sciences, Mashhad, Iran

**Keywords:** Reproduction number, Bayesian modeling, Tuberculosis, Iran

## Abstract

**Introduction:**

Tuberculosis (TB) is one of the most prevalent infectious diseases in the world, causing major public health problems in developing countries. The rate of TB incidence in Iran was estimated to be 13 per 100,000 in 2021. This study aimed to estimate the reproduction number and serial interval for pulmonary tuberculosis in Iran.

**Material and methods:**

The present national historical cohort study was conducted from March 2018 to March 2022 based on data from the National Tuberculosis and Leprosy Registration Center of Iran's Ministry of Health and Medical Education (MOHME). The study included 30,762 tuberculosis cases and 16,165 new smear-positive pulmonary tuberculosis patients in Iran. We estimated the reproduction number of pulmonary tuberculosis in a Bayesian framework, which can incorporate uncertainty in estimating it. Statistical analyses were accomplished in R software.

**Results:**

The mean age at diagnosis of patients was 52.3 ± 21.2 years, and most patients were in the 35–63 age group (37.1%). Among the data, 9121 (56.4%) cases were males, and 7044 (43.6%) were females. Among patients, 7459 (46.1%) had a delayed diagnosis between 1 and 3 months. Additionally, 3039 (18.8%) cases were non-Iranians, and 2978 (98%) were Afghans. The time-varying reproduction number for pulmonary tuberculosis disease was calculated at an average of 1.06 ± 0.05 (95% Crl 0.96–1.15).

**Conclusions:**

In this study, the incidence and the time-varying reproduction number of pulmonary tuberculosis showed the same pattern. The mean of the time-varying reproduction number indicated that each infected person is causing at least one new infection over time, and the chain of transmission is not being disrupted.

## 1 Introduction

Infectious diseases are caused by pathogens such as bacteria, viruses, parasites, or fungi that invade the body and multiply, leading to disease. Tuberculosis (TB) is one of the most prevalent infectious diseases in the world, posing a significant global health threat (Günther et al., 2023; Malani, 2010). *Mycobacterium tuberculosis* is the bacterium that causes TB, a highly contagious bacterial disease that mainly affects the lungs. TB spreads from person to person through the air when an infected person coughs, sneezes, or talks (Dartois & Rubin, 2022). According to the World Health Organization (WHO) report, TB is one of the top 13 causes of death worldwide and the leading cause of death by a single infectious agent. The disease is particularly prevalent in developing countries but also affects populations in developed countries (Chakaya et al., 2022; Organization WH, 2022; Petersen et al., 2022).

A total of 1.6 million people died from TB in 2021. The global TB epidemic affected 10.6 million individuals in total, including 6.2 million men, 3.4 million women, and 1.2 million children. People of all ages and from all nations are vulnerable to contracting tuberculosis. The 30 countries with high TB burdens accounted for 87% of new TB cases. Multidrug-resistant TB (MDR-TB) remains a public health crisis and a security threat. Only approximately one in three people with drug-resistant TB accessed treatment in 2020. Globally, half of TB-affected households face costs higher than 20% of their income, according to the latest national TB patient cost survey data. The world did not attain the goal of 0% tuberculosis patients by 2020, so households faced catastrophic costs due to TB disease. Although 98% of TB cases occur in low- and middle-income countries (LMICs), only a fraction of the necessary funding is allocated there. The United Nations' Sustainable Development Goals (SDGs) include the eradication of tuberculosis as a public health problem by the year 2030 (Chakaya et al., 2022; Organization WH, 2022; Petersen et al., 2022).

Iran, situated in the Eastern Mediterranean region (EMRO), has a population of approximately 85 million, including 3 million foreign nationals, primarily Afghan nationals constituting approximately 95% of this immigrant population (Khosravi & Dalvand, 2023). Iran shares its borders with Afghanistan and Pakistan, both designated by the WHO as high-burden TB countries. The Afghanistan-Iran-Pakistan border region (AIP region) has faced significant challenges stemming from conflicts, political and civil instability, large-scale displacements, droughts, and famines (Poureslami et al., 2004). These factors have contributed to the deteriorating health and quality of life of vulnerable populations in the area and resulted in elevated rates of communicable diseases, such as TB. TB is a significant health problem in Iran. According to the WHO report in 2021 on the epidemiological categorization of countries, territories, and areas based on incidence per 100,000 population in 2019, Iran ranked in the lower-moderate category (Bahraminia et al., 2021; Doosti et al., 2023a; Khademi & Sahebkar, 2021; Organization, 2021). Iran has implemented a TB control program since the 1970s, but the disease persists, particularly in disadvantaged and marginalized populations. The country faces several challenges in controlling TB, including limited access to diagnostics and treatment, stigma and discrimination, and poor infection control practices in healthcare settings (Azizi & Bahadori, 2011; Glaziou et al., 2015; Wilson et al., 2020).

One of the important factors in estimating the transmissibility and spread of an infectious disease is the basic reproduction number, denoted by $R_{0}$, which measures the average number of secondary cases generated by a single primary case in a population without immunity to the disease. The goal of public healthcare and epidemic control is to reach $R_{0}$ < 1(Delamater, Street, Leslie, Yang, & Jacobsen, 2019). Since $R_{0\;}$ should be calculated based on the spread of the disease in a completely susceptible (sensitive) population, the symbol $R_{0}$ is useful in initial calculations or estimates of the onset of an epidemic. Over time, as the number of susceptible individuals in the population decreases, the effective reproduction number (time-varying reproduction number) is denoted by $R_{t}\;\text{which}$ is used instead of $R_{0}$. The value of $R_{t}$ changes over time as the epidemic progresses. This change is usually due to an increase in the proportion of immune individuals due to recovery from a previous illness and is decreasing. In the long run, this trend leads to the value of $R_{t}$ becoming less than 1, resulting in the epidemic disappearing or the disease becoming endemic. The $R_{t}$ is a crucial epidemiological parameter that provides important information about the potential for an infectious disease to spread. $R_{t}$ is an important parameter for comprehending the transmission dynamics of infectious diseases (Koopman, 2004).

As a result of the outbreak of COVID-19, disruptions in TB services have led to an increase in the number of TB cases and TB-related fatalities worldwide, impacting the lives and livelihoods of millions of people worldwide. The COVID-19 pandemic has reversed a decade of progress in controlling the TB epidemic (Grassly & Fraser, 2008; Siettos & Russo, 2013). TB is the second leading cause of death from an infectious disease worldwide and requires an equal, if not greater, amount of attention as COVID-19 (Organization WH, 2022). In some countries, the incidence of TB has increased again due to neglect of control activities and a false sense of security resulting from the belief that previous control measures have had a lasting impact (Kamvar et al., 2019).

Bayesian estimation methods provide a powerful approach for estimating time-varying $R_{t}$ in infectious disease outbreaks. Bayesian methods use prior knowledge or beliefs about the distribution of $R_{t}$ together with observed data to update and refine those beliefs through the process of posterior inference. This approach yields estimates of $R_{t}$ that are more accurate and precise than traditional methods, which rely on assumptions about the underlying disease dynamics. Narula et al. applied the Bayesian molding technique to a deterministic model of TB to assess the situation of TB in Indian states and union territories. They estimated the value of R0 as 0.92, which shows that the epidemic of TB is unlikely to occur in India (Narula et al., 2015). As far as the researchers investigated, $R_{t}$ of pulmonary tuberculosis (PTB) in Iran has not been estimated, and there is no strong evidence in this regard. Therefore, the results of this study will be an important tool to help health policymakers develop more effective TB control measures. In recent years, many mathematical models have been developed to estimate $R_{t}$ during epidemic outbreaks, but there is no existing unique framework on this subject. This study aimed to estimate $R_{t}$ of smear-positive pulmonary tuberculosis (SPPTB) in Iran using the Bayesian framework.

## 2 Materials and methods

### 2.1 Design

From March 2018 to March 2022, a national historical cohort study was conducted in Iran using data from the National Tuberculosis and Leprosy Registration Center of Iran's MOHME. The study identified a total of 30,762 tuberculosis cases and 16,165 new smear-positive pulmonary tuberculosis patients across all 429 counties in Iran's 31 provinces. These cases were diagnosed by 61 medical universities.

### 2.2 Study participants

The study's selection criteria were centered on newly reported cases, specifically those with smear-positive pulmonary tuberculosis. These criteria were chosen because such cases are known to have a higher potential for disease transmission within the community and are a priority for treatment. On the other hand, the decision to exclude smear-negative PTB and extra-pulmonary tuberculosis (EXTB) cases from our study was driven by several factors, including concerns about diagnostic accuracy, data reliability, the need to focus on particular subpopulations and the goal of ensuring a consistent and uniform dataset. These exclusions were made to improve the quality and dependability of our analysis, ultimately leading to a more precise and accurate understanding of the specific subset of TB cases that were the focus of our investigation. The Tuberculosis and Leprosy Control Office of Iran's MOHME used a unique computerized questionnaire to register, analyze, and control TB morbidity, mortality, and related risk factors at the national level for all 429 counties in 31 provinces of Iran. The study extracted all demographic characteristics and other related risk factors, including age, pretreatment weight, height, sex, delayed diagnosis, location, bacilli density in the initial smear, nationality, prison condition, treatment outcomes of new PTB patients based on WHO definition, and duration of treatment. The date of onset of symptoms, date of diagnosis, and date of treatment were recorded for all patients.

### 2.3 Statistical analysis

The study summarized quantitative variables as the mean ± SD and qualitative variables as the frequency (%). The chi-square goodness-of-fit test was used to evaluate the uniform distribution of patients among the levels of qualitative variables. All preliminary data analyses were carried out using SPSS (version 22, Institute Inc., Chicago, IL, USA), R (version 4.2.1, www.r-project.org), and Microsoft Office Excel (version 2019) at a significance level of 0.05. Furthermore, Bayesian methodology was applied to estimate ${\;R}_{t}$.

### 2.4 Theory

Mathematical and computer models are used to understand patterns of infection spread in populations. These models vary from deterministic models of continuous populations to models of dynamically evolving contact networks between individuals. They provide insight, serve as scientific theories, help design studies, and help analyze data. To better understand and model the contagious dynamics, the impact of numerous variables ranging from the micro host-pathogen level to host-to-host interactions, as well as prevailing ecological, social, economic, and demographic factors across the globe, must be analyzed and thoroughly studied. Mathematical models have been developed to estimate several types of $R_{t}$ during epidemic outbreaks (Grassly & Fraser, 2008; Siettos & Russo, 2013).

#### 2.4.1 Estimating the time-varying reproduction number ($R_{t}$)

There are several methods for estimating $R_{t\;}$ based on epidemic data. Here, a few commonly used methods, such as the classical method for estimating $R_{t}$, during an outbreak of an infectious disease rely on the epidemic curve, which shows the number of new cases over time. This method assumes a fixed generation time, which is the time between the infection of a primary case and the infection of a secondary case. Using this assumption along with the epidemic curve, the classical method estimates $R_{t}$ at each point in time during the outbreak. Although the classical method is straightforward to implement, its assumption of a fixed generation time may not hold in all situations, which can lead to biased estimates of $R_{t}$. Furthermore, the method may not be suitable for outbreaks with rapidly changing transmission dynamics, such as those with the sudden implementation of control measures (Kamvar et al., 2019; Kenah et al., 2008).

The Wallinga and Teunis method is a technique to estimate $R_{t}$ during an outbreak of an infectious disease. This method allows for greater flexibility than the classical method in estimating $R_{t}$ (Wallinga & Lipsitch, 2007; Wallinga & Teunis, 2004).

The Bayesian method estimates $R_{t}$ using a Bayesian framework and assumes $R_{t}$ following a specific statistical model, such as a Gaussian process, which can incorporate uncertainty in the estimation of $R_{t}$ and provide credible intervals for $R_{t}$ estimates (Cori et al., 2013). This method can be used for both individual-level and aggregate-level data (Cori et al., 2020). Many studies tested their method on simulated outbreaks and found that the Bayesian method outperformed traditional methods in terms of accuracy and precision. They also applied the method to real-world outbreaks, including the 2009 H1N1 influenza pandemic and the 2014 Ebola outbreak, and showed that the Bayesian method provides more accurate and informative estimates of $R_{t}$ (Cauchemez et al., 2009; Cori et al., 2017; Donnelly et al., 2011). In this study, we estimated the $R_{t}$ within the Bayesian framework that is described in the following.

This method relies on a branching process model. In a branching process, each infected individual can produce a random number of secondary infections, and the total number of infections at any given time is the sum of all the infections produced by each infected individual.

The branching process $\left\{Y_{t};t\geq 0\right\}$ can be recursively defined using the representation

(1) $$Y_{t}=\sum_{j=1}^{Y_{t-1}}I_{t.j}\;.\;n=0.1.2.\ldots$$

Where $I_{n.j}$ can be interpreted as the number of new cases produced by the j th infected case in the t− th generation. The probability distribution of $I_{t.j}$ for all t and j is

$$P\left(I_{t.j}=k\right)=p_{k}\;k\geq 0$$

$$p_{k}=\frac{\text{exp}\left({-R}_{t}\right)R_{t}^{k}}{k!}k\geq 0$$

The sequence $\left\{p_{k}:k\geq 0\right\}$ is referred to as the offspring distribution. Most often, we do not have data on the number of infected individuals by each infectious but on the total number of infected individuals for a given time ($I_{t})$. The Poisson process in the branching process assumes that the number of new cases at any given time is a random variable that follows a Poisson distribution referred to as the offspring distribution, with the mean of this distribution being the product of $R_{t}$ and the sum of the past incidence $I_{t-s}$ of cases, weighted by $w_{s}$, the probability mass function of the generation time (the time between infection in a case and their infector). In practice, as the infection is difficult to observe, the incidence of symptomatic cases can be used instead, and $w_{s}$ can be approximated by the serial interval (SI) (Fraser, 2007; Nash et al., 2022).

The serial interval (SI), defined as the time interval between the onset of symptoms in a primary case and the onset of symptoms in a secondary case resulting from the primary case, is also a key factor in determining the speed and pattern of disease spread (Ten Asbroek et al., 1999; Vink et al., 2014; Vynnycky & White, 2010; Wallinga & Lipsitch, 2007).

TB has no early symptoms, and an infected person can easily spread the disease. The incubation period (no symptoms) for TB can vary widely, ranging from a few weeks to several months. On average, symptoms develop approximately 2–12 weeks after infection with TB bacteria. However, in some cases, symptoms may not appear until many months or even years later (Borgdorff et al., 2011).

In Iran, health centers have been gathering data on the incidence of TB among individuals who have been in close contact with an infected person since 2018, but the exact time of symptom onset in the second case is unknown. Estimating SI for TB disease can be challenging, particularly if the exact time of symptom onset in the second case is unknown.

It is worth noting that a significant proportion, approximately 85%, of individuals diagnosed with new smear-positive pulmonary tuberculosis (PTB) in Iran achieved a successful cure within a median duration of 6.33 months (with a confidence interval ranging from 6.31 to 7.2 months) (Nazar et al., 2021). This underscores that most PTB patients were effectively treated and monitored during these six months. It is important to recognize that the secondary cases identified in the study are primarily associated with this treatment duration. A sample of 436 primary cases was identified, while 601 secondary cases were reported for these primary cases. However, it is crucial to acknowledge the inherent difficulties posed by the extended latency period characteristic of tuberculosis. The challenge lies in the practicality of tracking all contacts of infectious cases over an extended period to ascertain whether they eventually develop the disease. This complexity arises due to tuberculosis's unique features and its protracted incubation period (Ma et al., 2020). Furthermore, it is essential to underscore that the data under examination in this study originate from a historical cohort and rely on registry-based records. These data sources have inherent limitations in capturing the entirety of the disease transmission dynamics accurately.

When data on the dates of symptom onset for secondary cases are not available, estimation of the serial interval can still be possible by relying on an assumption about the distribution of time intervals between symptom onset in primary and secondary cases. Parametric models for the SI, such as the gamma, Weibull, and log-normal distributions, are widely used to model infectious diseases (Cowling et al., 2009). Compared to the other distributions, the gamma distribution has a more flexible shape, which allows it to fit a wider range of SI distributions, including those with shorter or longer tails. The gamma distribution is also a more natural choice when SI is measured in discrete time units, such as days or weeks, as it models the probability of observing a certain number of days between the onset of symptoms in the primary and secondary cases. The Weibull and log-normal distributions are also similar to the gamma distribution in terms of empirical form (Firth, 1988). The gamma distribution is a continuous probability distribution that is frequently employed to model time intervals between events that occur independently at a constant rate. The estimation of the shape and scale parameters of the gamma distribution for the serial interval can be achieved using the method of uncertainty on the serial interval distribution as described in Cori et al. (Cori et al., 2013). This involves calculating the sample mean and variance of the time intervals between symptom onset in primary and secondary cases, then according to a truncated normal distribution, varying the mean and standard deviation of the serial interval. The process involves sampling pairs of means and standard deviations (μ and σ) while ensuring that the standard deviation is less than the mean. This constraint ensures that the probability density function of the Gamma distribution for the serial interval is zero at t = 0.

$R_{t}$ is a measure of the transmission potential of an infectious disease at a specific point in time. It is calculated as the ratio of the number of new infected cases at time t, denoted by $I_{t}$, and the total infection potential across all infected individuals at time t, denoted by $Y_{t}$. The model assumes that the incidence of new cases at time t. $I_{t}$ can be represented by a Poisson process:

(2) $$I_{t}∼Pois\left(R_{t}\sum_{s=1}^{t}I_{t-s}w_{s}\right)$$

The likelihood of observing the data (total cases $Y_{t}$ at time t) can be formulated as a product of Poisson probabilities:

$$L\left(Y_{t}|I_{0}.I_{1}\ldots .I_{t-1}.w_{s}.R_{t}\right)=\prod_{t}\left(\left(Y_{t}|Pois\left(R_{t}\sum_{s=1}^{t}I_{t-s}w_{s}\right)\right)\right)=\prod_{K=t-\tau }^{t}\frac{{\left({R_{t}Y}_{k}\left(w_{s}\right)\right)}^{I_{k}}\;\exp\left(-{R_{t}Y}_{k}\left(w_{s}\right)\right)}{I_{k}!}$$

The Gamma distribution for a and b can be formulated as a prior distribution for $R_{t}$:

$$P\left(R_{t}\right)=\frac{{R_{t}}^{a-1}\;\exp\left(-\frac{R_{t}}{b}\right)}{\Gamma \left(a\right)b^{a}}$$

The mean of this gamma distribution is $\frac{a}{b}$, and these parameters can be obtained from the serial interval distribution.

The posterior distribution for $R_{t}$ given the observed new cases $I_{t}$ and the prior information can be formulated as:

$$\left(R_{t}|I_{0}.I_{1}\ldots .I_{t-\tau -1}.I_{t-\tau .}I_{t-\tau +1}\ldots .I_{t}.w_{s}\right)$$

$$\propto L\left(Y_{t}|I_{0}.I_{1}\ldots .I_{t-1}.w_{s}.R_{t}\right)P\left(R_{t}\right)$$

$$=\left(\prod_{K=t-\tau }^{t}\frac{{\left({R_{t}Y}_{k}\left(w_{s}\right)\right)}^{I_{k}}\;\exp\left(-{R_{t}Y}_{k}\left(w_{s}\right)\right)}{I_{k}!}\right)\left(\frac{{R_{t}}^{a-1}\;\exp\left(-\frac{R_{t}}{b}\right)}{\Gamma \left(a\right)b^{a}}\right)$$

$$={R_{t}}^{a+\sum_{s=1}^{t}I_{k}-1}\;\exp\left(-R_{t}\left(\sum_{k=t-\tau }^{t}Y_{k}\left(w_{s}\right)+\frac{1}{b}\right)\right)\times \prod_{K=t-\tau }^{t}\frac{{\left(Y_{k}\left(w_{s}\right)\right)}^{I_{k}}}{I_{k}!}$$

Where *a* and *b* are the shape and scale parameters of the gamma-distributed prior for $R_{t}$. We used a gamma-distributed prior, conjugated to the Poisson likelihood, to obtain an analytical formulation of the posterior distribution of $R_{t}$. According to the expression above, the posterior distribution for $R_{t}$ given the incidence data, conditional on the SI distribution ${\;w}_{s}$, is a gamma distribution with the shape parameter $a+\sum_{s=1}^{t}I_{k}$ and the scale parameter $\frac{1}{\sum_{k=t-\tau }^{t}Y_{k}\left(w_{s}\right)+\frac{1}{b}}$.

We conducted a Bayesian analysis to estimate the mean of a branching process featuring a Poisson offspring distribution. Our primary objective was to ascertain the underlying mean parameter of this process while incorporating prior beliefs through the utilization of a gamma prior distribution. To estimate the posterior distribution for $R_{t}$, Bayesian inference techniques such as Markov chain Monte Carlo (MCMC) can be employed. The posterior distribution takes into account both the observed data $I_{t}$ and the prior information from the gamma distribution. We used the EpiEstim package to calculate $R_{t}$ based on the provided SI value. (Cori et al., 2013; McBryde et al., 2009; Nishiura & Chowell, 2014; Wallinga & Lipsitch, 2007).

#### 2.4.2 Comparative analysis of $R_{t}$ before and after the onset of the COVID-19 pandemic

To assess and compare $R_{t}$ values before and after the onset of the COVID-19 pandemic, a time series analysis was employed. This method was deemed essential due to the inherent temporal dependencies and the lack of data independence in the dataset. The analytical procedure involved several crucial steps. First, the data were meticulously structured into a time series format to capture temporal dynamics. Subsequently, visualizations were created to elucidate the $R_{t}$ patterns during both the pre-pandemic and post-pandemic periods. The selection of an appropriate time series model, such as ARIMA or VAR, was a pivotal step, followed by the separate estimation of model parameters for each period. Hypothesis testing was then conducted to discern statistically significant differences in $R_{t}$ values between the two periods, and the findings were interpreted accordingly. This approach sheds light on the impact of the COVID-19 pandemic on $R_{t}$ values, offering insights into the evolving dynamics of disease transmission.

## 3 Results

**Fig. 1:**
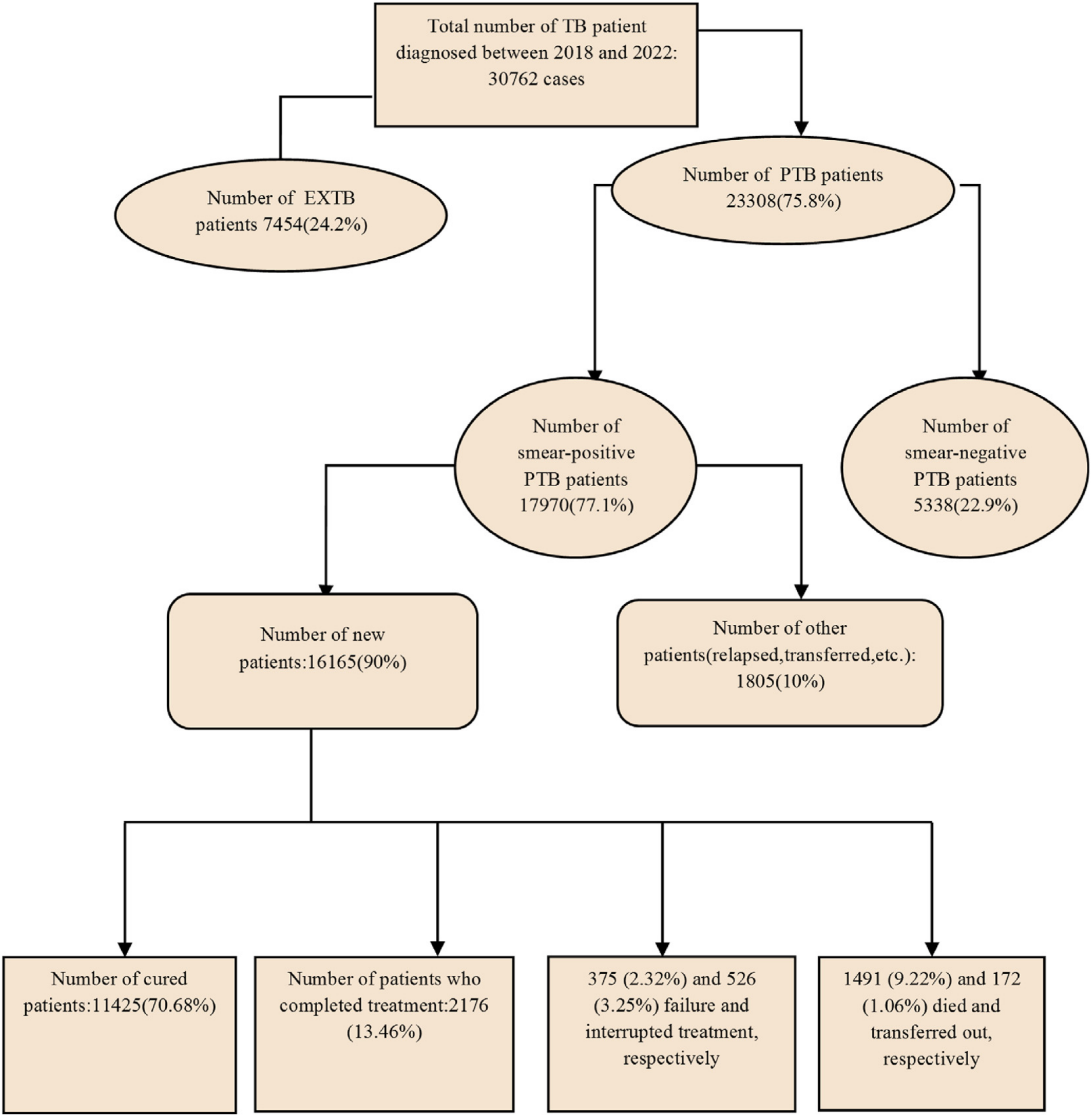
Flow diagram showing selection of the study population (tuberculosis (TB), pulmonary tuberculosis (PTB), extra-pulmonary tuberculosis (EXTB)).

Between March 2018 and March 2022, a total of 30762 active TB cases (PTB and EXTB) were diagnosed in Iran, 16165 of whom were new smear-positive PTB patients, and they were included in the study. Of the 16165 patients, 11425 (70.68%) were cured, 2176 (13.46%) completed the treatment period, 375 (2.32%) experienced treatment failure, 526 (3.25%) interrupted the treatment or failed to follow up, 1491 (9.22%) died, and 172 (1.06%) patients transferred out (Fig. 1).

A sample of 436 primary cases was identified, while 601 secondary cases were reported. Of the primary cases, 348 (79.8%) had only one secondary case, 52 (11.9%) had two secondary cases, and 36 (8.3%) had more than three secondary cases. On average, each primary case is associated with approximately 1.3 secondary cases. Additionally, 147 (33.7%) of the primary cases were of Afghan nationality, of which 195 secondary cases (32.5%) were reported.

The mean age at diagnosis of patients was 52.3 ± 21.2 years, and most patients were in the 35–63 years age group (37.1%). Among the data, 9121 (56.4%) cases were males, and 7044 (43.6%) were females. The patients’ height was defined in the TB registration system, but height for 772 cases and weight for 53 cases were not recorded, so body mass index (BMI) was calculated for 15,340 cases. A total of 5395 (35.3%) cases were underweight, 1892 (12.4%) were overweight, 701 (4.6%) were obese, and the others were in the normal range. Among patients, 7459 (46.1%) had a delayed diagnosis between 1 and 3 months. A total of 3039 (18.8%) cases had non-Iranian nationalities, and 2978 (98%) were Afghans (Table 1).

**Table 1:** Baseline characteristics and risk factors of new smear-positive pulmonary tuberculosis patients in Iran (n = 16,165).

Characteristics		New smear-positive PTB patients	P Value
Age (year)	Mean ± SD	52.3 ± 21.2	–
Age (year)	<15 (n %)	391(2.4)	<0.001 a
	15-35 (n %)	3944 (24.4)	
	36-63 (n %)	5990 (37.1)	
	>64 (n %)	5840 (36.1)	
Gender	Female (n %)	7044 (43.6)	<0.001 a
	Male (n %)	9121 (56.4)	
BMI (kg/m^2^)	Underweight (n %)	5395 (35.3)	<0.001 a
	Normal range (n %)	7312 (47.8)	
	Overweight (n %)	1892 (12.4)	
	Obese (n %)	701 (4.6)	
Bacilli density in initial smear	1–9 Basil (n %)	1501 (9.3)	<0.001 a
	1+ (n %)	5355 (33.1)	
	2+ (n %)	3456 (21.4)	
	3+ (n %)	5853 (36.2)	
Delayed diagnosis (month)	<1 (n %)	3865 (23.9)	<0.001 a
	1-3 (n %)	7459 (46.1)	
	3> (n %)	4841(29.9)	
Nationality	Iranian (n %)	13126 (81.2)	<0.001 a
	Others (n %)	3039 (18.8)	
Location	Urban (n %)	10948 (67.7)	<0.001 a
	Rural (n %)	5217 (32.3)	
Prison condition	Yes (n %)	473 (2.9)	<0.001 a
	No (n %)	15692 (97.1)	

a Significant at a level of 0.05.

**Fig. 2:**
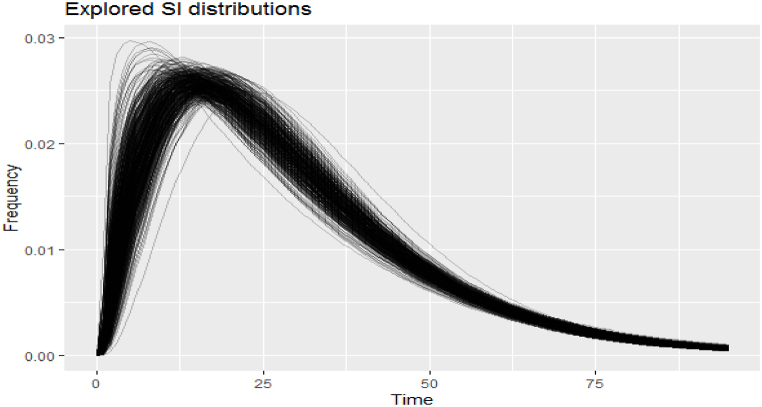
Serial interval of SPPTB in Iran from March 2018 to March 2022 weekly.

**Fig. 3:**
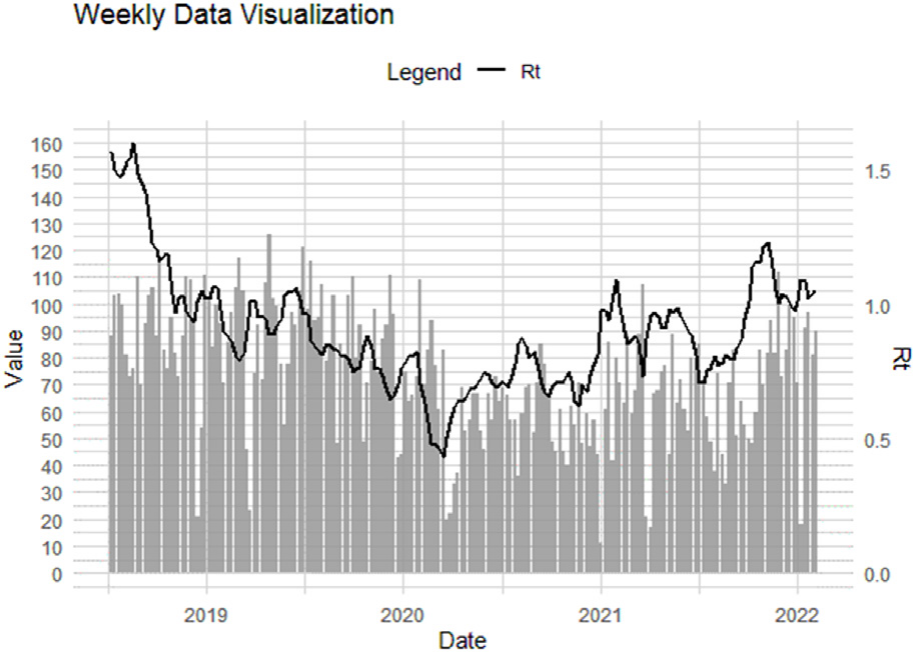
New cases and $R_{t}$ of SPPTB in Iran from March 2018 to March 2022 weekly.

Based on a sample of 436 primary cases and close contact data, the mean SI estimated approximately 29.6 weeks. In the Bayesian framework implemented with the EpiEstim package, uncertainty on the serial interval model is adopted, specifically using a gamma distribution with a mean of 29.6. This means that SI is assumed to follow a truncated normal distribution, varying the mean of the serial interval from 15 to 42 weeks with a mean value of 29.6 weeks. With this SI distribution in place, the Bayesian framework allows for the estimation of the posterior distribution for $R_{t}$. SI of SPPTB in Iran from 2018 to 2022 weekly is shown in Fig. 2. The weekly number of SPPTB cases and $R_{t}$ in Iran from March 2018 to March 2022 are shown in Fig. 3.

Table 2 provides a summary of the descriptive statistics for key epidemiological parameters related to SPPTB in Iran during the period from 2018 to 2022. The mean $R_{t}$ is reported as 1.06, with a standard deviation (SD) of 0.05, reflecting the average and variation in disease transmission. SI exhibits a mean of 29.59 weeks and an SD of 20.41 weeks. Quartile values, including the 1st quartile (0.88 for $R_{t}$ and 28.77 for SI) and 3rd quartile (1.88 for $R_{t}$ and 30.37 for SI), delineate the spread of data around the medians (1.02 for $R_{t}$ and 29.58 for SI). Notably, the minimum and maximum values for $R_{t}$ are 0.51 and 1.88, respectively, while those for SI are 25.49 and 32.96 weeks. Furthermore, the table presents the 95% credible intervals for $R_{t}$ (ranging from 0.96 to 1.15) and SI (from 18.58 to 31.43), offering insights into parameter uncertainty.

**Table 2:** Descriptive statistics of $R_{t}$ and SI of PTB disease in Iran during 2018–2022.

descriptive statistics	Mean ($R_{t}$)	SD ($R_{t}$)	Mean (SI) (week)	SD (SI) (week)
Minimum	0.51	0.03	25.49	18.02
1st Quartile	0.88	0.04	28.77	19.57
Median	1.02	0.05	29.58	20.35
Mean	1.06	0.05	29.59	20.41
3rd Quartile	1.88	0.05	30.37	21.26
Maximum	1.88	0.12	32.96	24.77
95%Crl	(0.96,1.15)		(18.58,31.43)	

Standard deviation (SD), 95% credible interval (95% Crl).

Table 3 displays the results of the time series analysis comparing $R_{t}$ values before and after the COVID-19 pandemic. Two separate ARIMA (0,1,1) models were employed for the pre-pandemic and post-pandemic periods. The analysis revealed distinct patterns $R_{t}$ before and after the pandemic. $R_{t}$ exhibited a decreasing trend during the pre-pandemic period, while the post-pandemic period witnessed an increasing trend. Specifically, the pre-pandemic ARIMA model had a coefficient_ma1 of 0.391, resulting in a lower $R_{t}$ mean. Conversely, the postpandemic ARIMA (0,1,1) model had a coefficient_ma1 of 0.422 and a positive drift term, contributing to a higher $R_{t}$ mean.

**Table 3:** **Time series** analysis comparing $R_{t}$ of PTB in Iran before the COVID-19 pandemic and after the COVID-19 pandemic.

Model	Coefficient_ma1	sigma2	Log_Likelihood	AIC	AICc	BIC	ME	RMSE
Prepandemic ARIMA	0.391	0.003	125.400	−244.800	−244.500	−237.510	0.000	0.054
Postpandemic ARIMA	0.422	0.003	147.580	−291.150	−291.030	−285.900	0.004	0.057

**Fig. 4:**
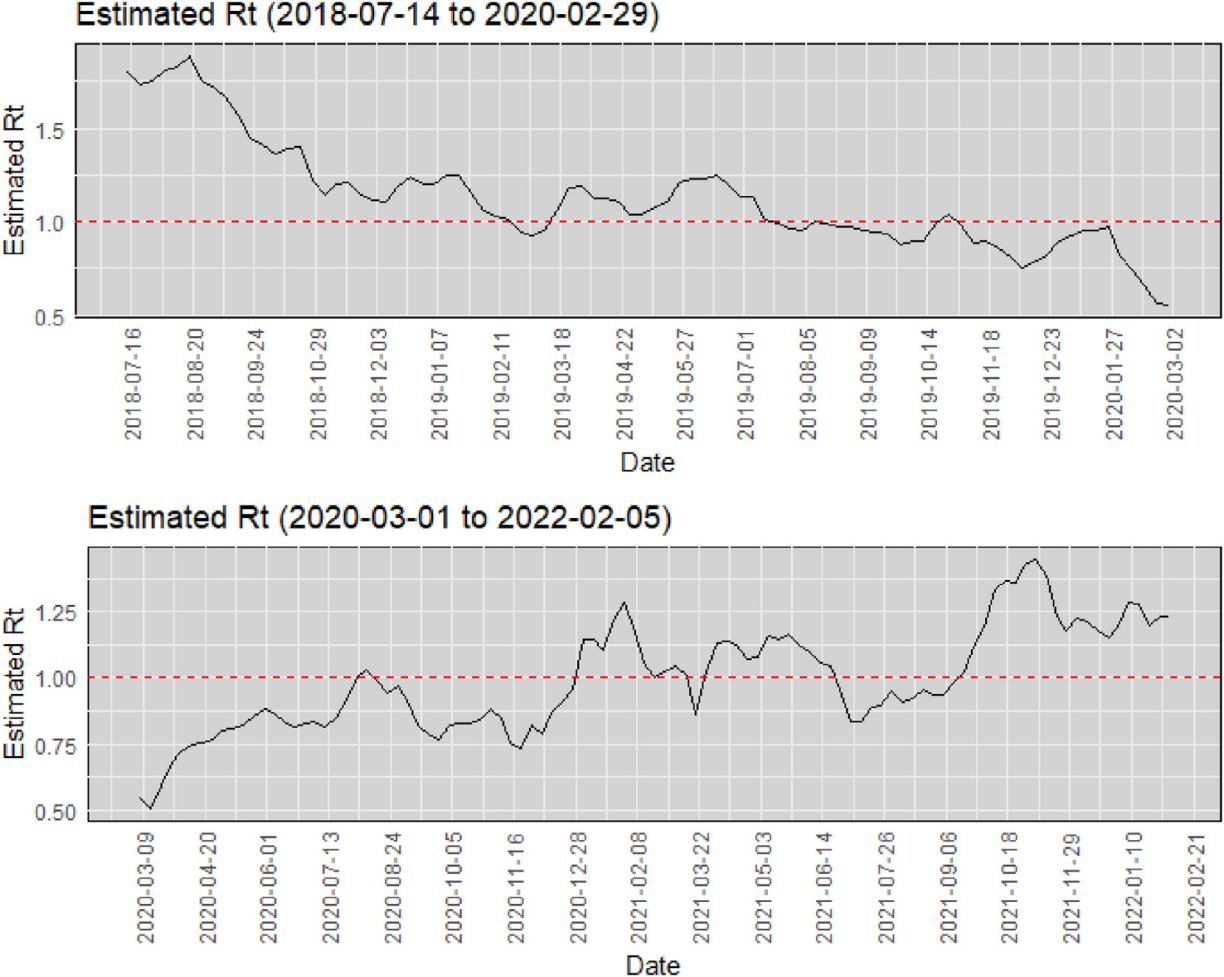
Comparison of $R_{t}$ time series trends before and after the COVID-19 pandemic.

In Fig. 4, we present a comparison of the time series trends of $R_{t}$ before and after the onset of the COVID-19 pandemic. These findings suggest that the COVID-19 pandemic has had an impact on $R_{t}$ dynamics in PTB transmission, with a shift from a decreasing trend to an increasing trend.

## 4 Discussion

The data for tuberculosis in Iran were examined, and it was found that between 2018 and 2022, a total of 30,762 cases were reported; in 2018 and 2019, 9000 cases were registered annually; in 2020 and 2021, approximately 6500 cases were registered annually; and in 2021 and 2022, a 27% decrease in TB was detected in Iran. One of the reasons for this reduction was attributed to the COVID-19 pandemic and the involvement of healthcare personnel in this emergency, leading to a reduction in tuberculosis screening in Iran. The COVID-19 pandemic has resulted in a significant reduction in TB detection and diagnosis worldwide, with some countries experiencing up to a 25% decrease in TB cases detected during the lockdown period. This is due to reduced access to health services, decreased TB screening, and the repurposing of TB testing equipment and staff for COVID-19 testing. The reduction in TB detection may lead to a higher risk of transmission and long-term implications for TB control (Husain et al., 2021; Rodrigues et al., 2022).

The time series analysis comparing $R_{t}$ values before and after the COVID-19 pandemic in the context of SPPTB transmission revealed notable differences. Before the pandemic, $R_{t}$ exhibited a decreasing trend, as indicated by the pre-pandemic ARIMA model with a lower mean $R_{t}$. In contrast, the post-pandemic period demonstrated an increasing $R_{t}$ trend, characterized by the post-pandemic ARIMA model with a higher mean $R_{t}$. This shift from a decreasing to an increasing trend suggests a significant impact of the COVID-19 pandemic on PTB transmission dynamics. The COVID-19 pandemic has profoundly impacted TB diagnosis and screening programs, posing significant challenges to the global effort to combat tuberculosis. The diversion of resources, reduced access to healthcare facilities, and disruptions in diagnostic services have hampered TB case finding and early detection during these unprecedented times. Additionally, the overlapping respiratory manifestations of tuberculosis and COVID-19 highlight the critical importance of understanding and managing the respiratory dynamics of these diseases to prevent transmission, ensure timely diagnosis, and develop effective treatment strategies (Cioboata et al., 2023).

The incidence rate of tuberculosis (TB) in Afghanistan and Pakistan, which are neighboring countries of Iran, is one of the highest in the world, estimated to be at least 40 cases per 100,000 people or greater (Organization, 2021). These countries have the highest prevalence of drug-resistant tuberculosis. According to the data, 147 (33.7%) of the primary cases were Afghan nationals who infected 195 (32.5%) secondary cases, indicating a high percentage. This suggests that immigrants from neighboring countries, particularly Afghanistan, can also have an impact on the trend of tuberculosis in Iran. Therefore, controlling the incidence of tuberculosis among immigrants and developing cohesive programs to follow up and treat these individuals can play a key role in eradicating the disease. According to a study conducted in Taiwan, female and young migrant workers from countries with high TB incidence were identified as significant sources of tuberculosis reservoirs. This finding suggests that latent tuberculosis infection (LTBI) may reactivate, leading to a probable risk of ongoing transmission during the first few years after their arrival in Taiwan (Lu et al., 2019). Borgdorff et al.'s study found that immigration from high TB prevalence areas may contribute to an increased risk of tuberculosis in Europe. The study focused on the transmission of tuberculosis between and within nationalities among residents of the Netherlands. The researchers used a transmission index to estimate the $R_{t}$ associated with recent transmission and found that 17% of Dutch TB cases were attributed to recent transmission from a non-Dutch source. The transmission index varied significantly by nationality, with the highest rates found among the Surinamese, Moroccan, and Turkish populations. These findings underscore the importance of targeted TB control measures for immigrant populations from high-prevalence areas to reduce the risk of ongoing transmission within the community. (Borgdorff et al., 1998).

Several studies have estimated $R_{t}$ for TB. For example, a study by Ma et al. used simulation studies to demonstrate that the cure model should be used when there is credible information on the percentage of individuals who will develop TB following infection. They estimated the SI for TB in the United States and Canada to be approximately 0.5 years and approximately 2.0 years in Brazil. This suggests a higher occurrence of reinfected TB in developing countries such as Brazil (Ma et al., 2020). Another study by Salpeter et al. estimated $R_{t}$ to be approximately 0.55 (Salpeter & Salpeter, 1998). Another study by Zhao, Li, and Yuan used the SEIR epidemic model with age groupings and seniors to investigate the role of age in the transmission of tuberculosis in mainland China from 2005 to 2016. Then, they evaluated the parameters by the least squares method and simulated the model, and they estimated an $R_{0}$ of 1.79, with a 95% confidence interval for $R_{0}$ of (1.78, 1.80) by Latin hypercube sampling (Zhao et al., 2017). There was no study to estimate $R_{t}$ and SI for TB in Iran. In this study, the SPPTB incidence and $R_{t}$ follow similar patterns. $R_{t}$ fluctuates approximately 1, occasionally dips below it and occasionally rises above it, but the average remains at approximately 1.06. The mean value of $R_{t}$ provides us with insights into potential future trends in SPPTB incidence. This suggests that there may be an increase in SPPTB cases in the future, and this information can be valuable in understanding the likely trajectory of the disease. We have concluded that the chain of transmission has not been interrupted (Dietz, 1993). In recent decades, the incidence of the disease has been gradually reduced in Iran. However, the reduction in the incidence of the disease has stopped in the country in recent years. This could be due to an increase in immigration, diabetes, HIV/AIDS, and the prevalence of drug-resistant strains. In conclusion, the increase in predisposing risk factors for catching TB, especially migration and Beijing strain, shows that in the absence of accurate monitoring, TB cases will increase in the near future in Iran (Fadaee et al., 2020). Despite TB treatment success and the low prevalence of MDR cases, TB incidence has not decreased significantly in Iran. Delays in diagnosis, high TB burden in refugees, and close contact investigation and prophylaxis are important issues in the TB control program in Iran to be considered in control planning (Doosti et al., 2023b).

One strength of the Bayesian estimation method for pulmonary tuberculosis $R_{t}$ using new smear-positive cases in Iran is that it allows for a more precise estimation of the $R_{t}$ variable over time, which is critical for understanding the transmission dynamics of the SPPTB in Iran. The study also used a large dataset of SPPTB cases from the Iranian National Tuberculosis Registry, which helped to ensure that the results were robust and representative of the broader population. However, there are also several limitations to consider. First, the study only covered the period from 2018 to 2022, which may not represent longer-term trends in SPPTB transmission in Iran. Second, the study relied on assumptions about the distribution of the SI, which could introduce some uncertainty into the results. It should be noted that the value of $R_{t}$ may be subject to overestimation due to an imprecise calculation of SI and a lack of accurate knowledge about the incubation time of TB. Additionally, there is a possibility of underestimation due to decreased tuberculosis case detection during the years 2020–2022 as a result of the strain on healthcare systems caused by the COVID-19 pandemic. Despite these limitations, the Bayesian estimation method used in this study provides a valuable tool for estimating $R_{t}$ of SPPTB in Iran, which could inform public health strategies aimed at controlling the spread of the disease. Future studies could build on this work by incorporating additional sources of data and extending the analysis to cover longer periods.

## 5 Conclusion

The findings of this study shed light on the patterns of TB transmission and underscore the significance of collecting and examining temporal data related to the disease, including $R_{t}$. Such insights hold the potential to guide health policymakers in crafting interventions aimed at disease control. Our research adds to the collective endeavor to combat and eradicate TB, a persistent global public health concern. Enhanced comprehension of TB transmission patterns equips us to devise more efficient approaches to prevention, diagnosis, and treatment, leading to better health outcomes for individuals and communities impacted by TB.

Based on this study, it appears that there has been a consistent and constant trend in both the weekly incidence of SPPTB and $R_{t}$ of SPPTB in Iran. Moreover, this suggests that due to the COVID-19 pandemic, there may have been insufficient efforts to address and reduce the spread of this disease, ultimately preventing its eradication from the country.

## Ethics approval and consent to participate

The study was approved by the Research Ethics Committee of Mashhad University of Medical Sciences, and all methods were carried out following relevant guidelines and regulations. Informed consent was waived by the Research Ethics Committee of Mashhad University of Medical Sciences, Code: IR.MUMS.FHMPM.REC.1401.052.

## Availability of data and materials

The dataset used in this study is available on request from the corresponding author. Due to confidentiality concerns, the data are not publicly available. Requests for the data will be considered on a case-by-case basis and will be subject to approval from the Ministry of Health of Iran.

## Consent for publication

Not Applicable.

## Funding

This study was financially supported by the Vice-chancellor for Research and Technology, Mashhad University of Medical Sciences, Iran (project No. 4001759).

## CRediT authorship contribution statement

**Maryam Rastegar:** Writing – original draft, Software, Methodology, Formal analysis. **Eisa Nazar:** Writing – review & editing, Data curation. **Mahshid Nasehi:** Writing – review & editing, Data curation. **Saeed Sharafi:** Data curation. **Vahid Fakoor:** Writing – review & editing, Supervision, Methodology. **Mohammad Taghi Shakeri:** Supervision, Project administration.

## Declaration of competing interest

The authors declare that they have no conflicts of interest.

## Abbreviations

TB: Tuberculosis
PTB: Pulmonary tuberculosis
SPPTB: Smear-positive pulmonary tuberculosis
EXTB: Extra-pulmonary tuberculosis
$R_{t}$: Effective reproduction number
SI: Serial Interval

## References

[bib1] Azizi M.H., Bahadori M. (2011). A brief history of tuberculosis in Iran during the 19th and 20th centuries. Archives of Iranian Medicine.

[bib2] Bahraminia F., Azimi T., Zangiabadian M., Nasiri M.J., Goudarzi M., Dadashi M., Fooladi A.A.I. (2021). Rifampicin-resistant tuberculosis in Iran: A systematic review and meta-analysis. Iranian Journal of Basic Medical Sciences.

[bib3] Borgdorff M.W., Nagelkerke N., Van Soolingen D., De Haas P.E., Veen J., Van Embden J.D. (1998). Analysis of tuberculosis transmission between nationalities in The Netherlands in the period 1993–1995 using DNA fingerprinting. American Journal of Epidemiology.

[bib4] Borgdorff M.W., Sebek M., Geskus R.B., Kremer K., Kalisvaart N., van Soolingen D. (2011). The incubation period distribution of tuberculosis estimated with a molecular epidemiological approach. International Journal of Epidemiology.

[bib5] Cauchemez S., Ferguson N.M., Wachtel C., Tegnell A., Saour G., Duncan B., Nicoll A. (2009). Closure of schools during an influenza pandemic. The Lancet Infectious Diseases.

[bib6] Chakaya J., Petersen E., Nantanda R., Mungai B.N., Migliori G.B., Amanullah F. (2022). The WHO Global Tuberculosis 2021 Report–not so good news and turning the tide back to End TB. International Journal of Infectious Diseases.

[bib7] Cioboata R., Biciusca V., Olteanu M., Vasile C.M. (2023). COVID-19 and tuberculosis: Unveiling the dual threat and shared solutions perspective. Journal of Clinical Medicine.

[bib8] Cori A., Cauchemez S., Ferguson N.M., Fraser C., Dahlqwist E., Demarsh P.A. (2020).

[bib9] Cori A., Donnelly C.A., Dorigatti I., Ferguson N.M., Fraser C., Garske T. (2017). Key data for outbreak evaluation: Building on the Ebola experience. Philosophical Transactions of the Royal Society B: Biological Sciences.

[bib10] Cori A., Ferguson N.M., Fraser C., Cauchemez S. (2013). A new framework and software to estimate time-varying reproduction numbers during epidemics. American Journal of Epidemiology.

[bib11] Cowling B.J., Fang V.J., Riley S., Peiris J.M., Leung G.M. (2009). Estimation of the serial interval of influenza. Epidemiology.

[bib12] Dartois V.A., Rubin E.J. (2022). Anti-tuberculosis treatment strategies and drug development: Challenges and priorities. Nature Reviews Microbiology.

[bib52] Delamater PL, Street EJ, Leslie TF, Yang YT, Jacobsen KH (2019). Complexity of the basic reproduction number (R0). Emerging infectious diseases.

[bib13] Dietz K. (1993). The estimation of the basic reproduction number for infectious diseases. Statistical Methods in Medical Research.

[bib14] Donnelly C.A., Finelli L., Cauchemez S., Olsen S.J., Doshi S., Jackson M.L. (2011). Serial intervals and the temporal distribution of secondary infections within households of 2009 pandemic influenza A (H1N1): Implications for influenza control recommendations. Clinical Infectious Diseases.

[bib15] Doosti A., Nasehi M., Moradi G., Roshani D., Sharafi S., Ghaderi E. (2023). The pattern of tuberculosis in Iran: A national cross-sectional study. Iranian Journal of Public Health.

[bib16] Doosti A., Nasehi M., Moradi G., Roshani D., Sharafi S., Ghaderi E. (2023). The pattern of tuberculosis in Iran: A national cross-sectional study. Iranian Journal of Public Health.

[bib17] Fadaee M., Rashedi J., Arabi S., Poor B.M., Kafil H.S., Pourostadi M. (2020). Stopping of the downtrend of Tuberculosis in Iran, a systematic review of associated risk factors. Infectious Disorders: Drug Targets.

[bib18] Firth D. (1988). Multiplicative errors: Log-normal or gamma?. Journal of the Royal Statistical Society: Series B.

[bib19] Fraser C. (2007). Estimating individual and household reproduction numbers in an emerging epidemic. PLoS One.

[bib20] Glaziou P., Sismanidis C., Floyd K., Raviglione M. (2015). Global epidemiology of tuberculosis. Cold Spring Harbor Perspectives in Medicine.

[bib21] Grassly N.C., Fraser C. (2008). Mathematical models of infectious disease transmission. Nature Reviews Microbiology.

[bib22] Günther G., Guglielmetti L., Leu C., Lange C., van Leth F., Hafizi H. (2023). Availability and costs of medicines for the treatment of tuberculosis in Europe. Clinical Microbiology and Infection.

[bib23] Husain A.A., Monaghan T.M., Kashyap R.S. (2021). Impact of COVID-19 pandemic on tuberculosis care in India. Clinical Microbiology and Infection.

[bib24] Kamvar Z.N., Cai J., Pulliam J.R., Schumacher J., Jombart T. (2019). Epidemic curves made easy using the R package incidence. F1000Research.

[bib25] Kenah E., Lipsitch M., Robins J.M. (2008). Generation interval contraction and epidemic data analysis. Mathematical Biosciences.

[bib26] Khademi F., Sahebkar A. (2021). An updated systematic review and meta-analysis on Mycobacterium tuberculosis antibiotic resistance in Iran (2013-2020). Iranian Journal of Basic Medical Sciences.

[bib27] Khosravi R., Dalvand F. (2023). Subjective well-being of Afghan immigrants residing in shiraz and its correlates. Journal of Population Association of Iran.

[bib28] Koopman J. (2004). Modeling infection transmission. Annual Review of Public Health.

[bib30] Lu C.-W., Lee Y.-H., Pan Y.-H., Chang H.-H., Wu Y.-C., Sheng W.-H., Huang K.-C. (2019). Tuberculosis among migrant workers in Taiwan. Globalization and Health.

[bib31] Ma Y., Jenkins H.E., Sebastiani P., Ellner J.J., Jones-López E.C., Dietze R. (2020). Using cure models to estimate the serial interval of tuberculosis with limited follow-up. American Journal of Epidemiology.

[bib32] Malani P.N. (2010). Mandell, Douglas, and Bennett's principles and practice of infectious diseases. JAMA.

[bib33] McBryde E., Bergeri I., van Gemert C., Rotty J., Headley E., Simpson K. (2009). Early transmission characteristics of influenza A (H1N1) v in Australia: Victorian state, 16 May–3 June 2009. Euro Surveillance.

[bib34] Narula P., Azad S., Lio P. (2015). Bayesian melding approach to estimate the reproduction number for tuberculosis transmission in Indian states and union territories. Asia-Pacific Journal of Public Health.

[bib35] Nash R.K., Nouvellet P., Cori A. (2022). Real-time estimation of the epidemic reproduction number: Scoping review of the applications and challenges. PloS Digital Health.

[bib36] Nazar E., Baghishani H., Doosti H., Ghavami V., Aryan E., Nasehi M. (2021). Bayesian spatial survival analysis of duration to cure among new smear-positive pulmonary tuberculosis (PTB) patients in Iran, during 2011–2018. International Journal of Environmental Research and Public Health.

[bib37] Nishiura H., Chowell G. (2014). Early transmission dynamics of Ebola virus disease (EVD), west Africa, March to August 2014. Euro Surveillance.

[bib38] Organization W.H. (2021).

[bib39] Organization WH (2022).

[bib40] Petersen E., Al-Abri S., Chakaya J., Goletti D., Parolina L., Wejse C. (2022). World TB Day 2022: Revamping and reshaping global TB control programs by advancing lessons learnt from the COVID-19 pandemic. International Journal of Infectious Diseases.

[bib41] Poureslami I.M., MacLean D.R., Spiegel J., Yassi A. (2004). Sociocultural, environmental, and health challenges facing women and children living near the borders between Afghanistan, Iran, and Pakistan (AIP region). Medscape General Medicine.

[bib42] Rodrigues I., Aguiar A., Migliori G.B., Duarte R. (2022). Impact of the COVID-19 pandemic on tuberculosis services. Pulmonology.

[bib43] Salpeter E.E., Salpeter S.R. (1998). Mathematical model for the epidemiology of tuberculosis, with estimates of the reproductive number and infection-delay function. American Journal of Epidemiology.

[bib44] Siettos C.I., Russo L. (2013). Mathematical modeling of infectious disease dynamics. Virulence.

[bib45] Ten Asbroek A.H., Borgdorff M.W., Nagelkerke N., Devillé W., van Embden J., van Soolingen D. (1999). Estimation of serial interval and incubation period of tuberculosis using DNA fingerprinting. International Journal of Tuberculosis & Lung Disease.

[bib46] Vink M.A., Bootsma M.C.J., Wallinga J. (2014). Serial intervals of respiratory infectious diseases: A systematic review and analysis. American Journal of Epidemiology.

[bib47] Vynnycky E., White R. (2010).

[bib48] Wallinga J., Lipsitch M. (2007). How generation intervals shape the relationship between growth rates and reproductive numbers. Proceedings of the Royal Society B: Biological Sciences.

[bib49] Wallinga J., Teunis P. (2004). Different epidemic curves for severe acute respiratory syndrome reveal similar impacts of control measures. American Journal of Epidemiology.

[bib50] Wilson A.L., Courtenay O., Kelly-Hope L.A., Scott T.W., Takken W., Torr S.J., Lindsay S.W. (2020). The importance of vector control for the control and elimination of vector-borne diseases. PLoS Neglected Tropical Diseases.

[bib51] Zhao Y., Li M., Yuan S. (2017). Analysis of transmission and control of tuberculosis in Mainland China, 2005–2016, based on the age-structure mathematical model. International Journal of Environmental Research and Public Health.

