# Supplementary material for: Bayesian estimation of the time-varying reproduction number for pulmonary tuberculosis in Iran: A registry-based study from 2018 to 2022 using new smear-positive cases
Source: Infect Dis Model. 2024 May 10;9(3):963–74. doi: 10.1016/j.idm.2024.05.003 (PMC11169078; doi:10.1016/j.idm.2024.05.003)
Supplement: Multimedia component 1 [file mmc1.docx]

**R script**

library(EpiEstim)

library(ggplot2)

library(MASS)

library(readxl)

library(incidence)

## load data

TB2022 <- read_excel("g:/TB/TB2022.xlsx")

View(TB2022)

# Importing the excel workbook for

# checking the number of sheets it contains

excel_sheets("g:/TB/TB2022.xlsx")

incidence<-read_excel("g:/TB/TB2022.xlsx",sheet="incidence",col_names = TRUE)

incidence

head(incidence)

## serial interval (SI) distribution:

dates <- incidence$dates

if (!all(class(dates) %in% c("Date", "numeric"))) {

print("Dates must be of class Date or numeric")

}

dist<-"G"

config <- make_config(list(si_parametric_distr = dist, mean_si = 29.6, std_mean_si =1.8,

min_mean_si = 15, max_mean_si =42,

std_si = 20, std_std_si =1.2,

min_std_si = 6.5, max_std_si =20))

res_uncertain_si <- estimate_R(incidence$I,

method = "uncertain_si",

config = config)

#> Default config will estimate R on weekly sliding windows.

#> To change this change the t_start and t_end arguments.

plot(res_uncertain_si, "SI")

res_uncertain_si

plot(res_uncertain_si,X=dates)

summary(res_uncertain_si$R)

summary(res_uncertain_si$SI)
